# Integrated Non-targeted and Targeted Metabolomics Uncovers Amino Acid Markers of Oral Squamous Cell Carcinoma

**DOI:** 10.3389/fonc.2020.00426

**Published:** 2020-04-15

**Authors:** Xi-Hu Yang, Yue Jing, Shuai Wang, Feng Ding, Xiao-Xin Zhang, Sheng Chen, Lei Zhang, Qin-Gang Hu, Yan-Hong Ni

**Affiliations:** ^1^Central Laboratory of Stomatology, Nanjing Stomatological Hospital, Medical School of Nanjing University, Nanjing, China; ^2^Department of Oral and Maxillofacial Surgery, Nanjing Stomatological Hospital, Medical School of Nanjing University, Nanjing, China; ^3^Department of Oral and Maxillofacial Surgery, Affiliated Hospital of Jiangsu University, Zhenjiang, China; ^4^Department of Oral Pathology, Nanjing Stomatological Hospital, Medical School of Nanjing University, Nanjing, China

**Keywords:** oral squamous cell carcinoma, non-targeted metabolomics, targeted metabolomics, amino acid markers, diagnosis

## Abstract

**Purpose:** It is very important to develop potential molecular associated with oral squamous cell carcinoma (OSCC) malignant transformation and progression. Thus, the aim of our study was to determine the amino acid metabolic characteristics of OSCC patients and test their diagnostic value.

**Experimental Design:** Eight pairs of matched tumor and normal samples were collected for gas chromatography–mass spectrometry (GC-MS) high-throughput untargeted analysis. Another 20 cases (each case including tumor and normal tissues) were also enrolled for ultrahigh-performance liquid chromatography–tandem mass spectrometer (UHPLC-MS/MS) amino acid quantitative analysis. *T*-test and receiver operating characteristic (ROC) curve analysis were used to determine candidate markers. Principal component analysis, partial least squares discriminant analysis, and heat map analysis were used to verify the ability of candidate markers to distinguish tumors from normal tissues.

**Results:** A total of 10 amino acids biomarker were selected as OSCC candidate diagnostic biomarkers by GC-MS high-throughput untargeted metabolomics analyses [area under the curve (AUC) >0.80]. We further measured the specific concentration of these candidate amino acids biomarkers in another batch of 20 cases by UHPLC-MS/MS quantitative analysis. The result validated that nine amino acids had been detected, which had statistically significant difference (*t*-test, *p* < 0.05). Moreover, three of nine amino acid markers (glutamate, aspartic acid, and proline) displayed high sensitivity and specificity (AUC >0.90) by ROC curve analysis and obtained optimal sensitivity and specificity by binary logistic regression in the Glmnet package (AUC = 0.942).

**Conclusions:** In conclusion, a panel including three amino acids (glutamate, aspartic acid, and proline) was identified as potential diagnostic biomarkers of OSCC by a combination of non-targeted and targeted metabolomics methods.

## Introduction

Oral squamous cell carcinoma (OSCC) is one of the most common malignant tumors in the head and neck region. Oral squamous cell carcinoma kills 6 million people worldwide every year (1); tobacco use, alcohol consumption, and human papillomavirus infection are major risk factors of oral cancer (2). Auxiliary methods for the diagnosis of oral cancer include physical examination, histopathological examination of tissue biopsies, endoscopy, computed tomography, and magnetic resonance imaging. Although diagnostic methods for OSCC have been greatly improved, survival rate remains poor due to regional and distant metastases (3).

Dysregulation in metabolic pathways was observed in almost all tumors, and the most striking feature of cancer cells is that they alter their metabolic pathways to meet cancer cell energy need. Changes in cell metabolism are benefit of tumor development. Cell metabolic phenotypes can be used to image tumors and predict patient's outcomes (4). It is very important to study the metabolic mechanism of oral cancer progression, which will help improve the diagnosis and treatment of oral cancer. Many studies have confirmed that OSCC tissues undergo significant metabolic changes compared to normal tissues, such as lactate, glycine, proline, and hydroxyproline, 3AMP, uracil, spermidine, and c-glycosyltryptophan, 2-hydroxyglutarate, and glycerol-3-monophosphate high expression in tumor tissues (21, 27). The metabolic pathways associated with oral cancer mainly include glycolysis, amino acid metabolism and pentose phosphate pathway, and RNA biosynthesis. It had been reported that cancer cells are able to gain energy from lactic acid fermentation, even when oxygen is in plentiful supply, known as the Warburg effect (5, 6). At the same time, cancer cells can get energy from other metabolic pathways, such as amino acid and lipid metabolism (7–9).

For example, glutaminolysis, deposing glutamine into glutamate, further α-ketoglutarate for maintaining tricarboxylic acid cycle, replenishes glucose metabolism and provides energy for cancer cells. It has been reported that the oncogene c-myc can regulate glutaminolysis catabolism (7).

Therefore, we concluded that metabolic changes in tumor cells were regulated by related gene.

In a word, it is urgent to develop a panel of useful biomarkers for OSCC early diagnosis (10, 11). However, metabolomics studies of OSCC are very limited up to date; many studies use serum and saliva samples (12, 13), and there is lack of research on a specific metabolic type, such as amino acid metabolism. In this study, we aim to explore tissue-based amino acids metabolite biomarkers of OSCC by untargeted and targeted metabolomics, which not only distinguish cancer cell from normal tissues, but also used as prognostic methods for early detection of OSCC.

## Materials and Methods

### Tumor Tissue Specimens

**Figure 1:**
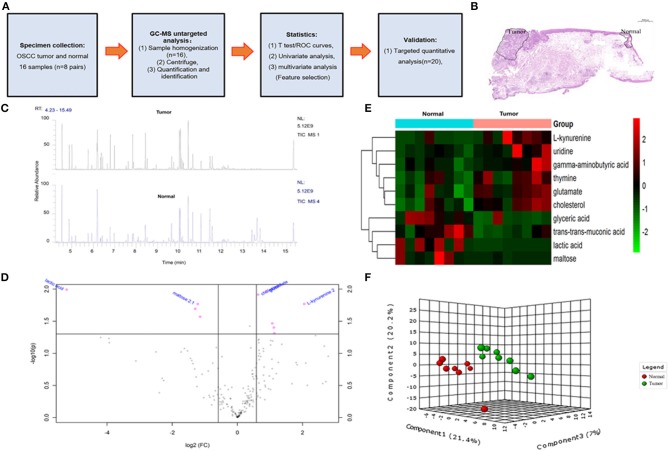
The overall design idea of this experiment and preliminary screening of differential metabolite in untargeted group. **(A)** Research flowchart: including specimen collection, GC-MS analysis untargeted analysis, data analysis, and UHPLC-MS targeted validation. **(B)** Schematic diagram of sample collection site, tumor, and normal tissue. **(C)** Mass spectrometry of the tumor and normal tissue. **(D)** Volcano plot analysis between tumor and normal by *t*-test. **(E)** Comparison of tumor and normal using normalized intensities of 10 significance metabolites. The mean value of 10 metabolites in each group was obtained, and then *Z*-score transformation generates heat map. **(F)** Scores plot segregated tumor and normal by PLS-DA analysis in untargeted group.

This study was reviewed and approved by the medical ethics committee of Nanjing Stomatological Hospital, following the Declaration of Helsinki. All cases included in the study were between 30 and 70 years old and had signed informed consent forms. Matching of collected of tumor and normal samples (eight pairs, *n* = 16) was performed by gas chromatography–mass spectrometry (GC-MS) high-throughput untargeted analysis. Another 20 paired cases (each case including tumor and normal tissues, Figures 1A,B) were also enrolled in this study for ultrahigh-performance liquid chromatography–tandem mass spectrometer (UHPLC-MS/MS) amino acid targeted quantitative analysis. All fresh tissues were snap-frozen in liquid nitrogen within 30 min after operation and stored at −80°C freezer until they were processed.

### GC-MS Untargeted Analysis

The GC-MS experiment was carried out in Nanjing University of Chinese Medicine and was strongly supported by Associate Professor Xie Tong. The materials needed for the experiment were prepared by referring to the previous methods (14). The specific operation steps are shown in reference.

### UHPLC-MS/MS Targeted Quantitative Analysis

Ultrahigh-performance liquid chromatography–tandem mass spectrometer targeted quantitative analysis specific operation steps refer to our previous report (15). The UHPLC separation was carried out using an Agilent 1290 Infinity II series UHPLC System (Agilent Technologies, California, USA), equipped with a Waters ACQUITY UPLC BEH Amide column (Waters Corporation, USA) (100 × 2.1 mm, 1.7 μm). Mobile phase A was 1% formic acid in water, and mobile phase B was 1% formic acid in acetonitrile. The column temperature and auto-sampler temperature were set at 35 and 4°C, respectively. An AJS–electrospray ionization interface was connected with Agilent 6460 triple quadrupole mass spectrometer (Agilent Technologies) for assay development. The MRM parameters of the target analytes are controlled by flowing injection of the standard solution of a single analyte.

### Metabolomics Data Processing

For GC-MS raw data search and determination, see our previous report. Peak area ratios of metabolites in each sample were calculated with Xcalibur 2.2 (Thermo Scientific, Massachusetts, USA) by normalization to the internal standard. Specific concentrations of individual metabolites can be obtained directly by UHPLC-MS/MS analysis (nmol/g).

### Statistical Analysis

Our metabonomics statistical analyses were performed using the MetaboAnalyst (https://www.metaboanalyst.ca). In the GC-MS untargeted group, the bucketed experimental data were normalized by the total spectral intensity, in addition to Pareto scale (for multivariate analysis). Differential amino acid metabolites were determined by volcano plot with fold-change threshold (>1.5) and *t*-test threshold (<0.05). Then, the differential metabolites were further analyzed by receiver operating characteristic (ROC) curve to determined candidate biomarkers. Univariate analysis was by Student *t*-test with (false discovery rate–adjusted *p*-value of 0.05). Multivariate analysis included unsupervised principal component analysis (PCA) and partial least squares discriminant analysis (PLS-DA). Pheatmap package was used to carry hierarchical cluster analysis.

In the UHPLC-MS/MS targeted group, the correlation coefficients of all target analytes were >0.9959, indicating that a good quantitative analysis result was obtained, which was satisfying for targeted metabolomics analysis. For specific steps and technical parameters, refer to our previous report (15). The statistical difference of quantitative analysis results was analyzed by GraphPad Prism 8 (GraphPad Software corporation, California, USA) (*t*-test, *p* < 0.05).

## Results

### Clinical Characteristics of the Patients in This Study

The overall flowchart of this study is shown in Figure 1A. A total of 28 cases were included in the study, eight cases (contain pair tumor and normal tissues) of which were used for GC-MS high-throughput untargeted analysis, and another 20 cases (contain pair tumor and normal tissues) were used for UHPLC-MS amino acid targeted quantitative analysis.

A total of 10 amino acids were selected as OSCC candidate diagnostic biomarkers by GC-MS untargeted metabolomics analyses.

**Figure 2:**
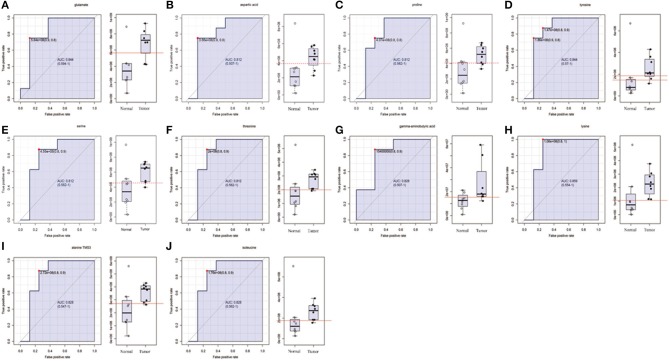
The list of amino acids selected as candidate tumor biomarkers by ROC curve analysis (AUC >0.80) in GC-MS untargeted group. **(A–J)** Individual amino acid ROC curves. AUC (0.5–0.7), low accuracy; AUC (0.7–0.9), moderate accuracy; AUC (>0.9), high accuracy.

In order to determine potential metabolic markers of the OSCC, we first performed GC-MS high-throughput untargeted metabolomics analysis for eight matched pairs (*n* = 16) of OSCC (Figures 1B,C). The metabolites identified included amino acids, carbohydrate, lipids, and other compounds. All metabolites were first analyzed by volcano plot with fold-change threshold (>1.5) and Student *t*-test threshold (*p* < 0.05). Ten metabolites were screened out (fold change >1.5 or <0.5 and *p* < 0.05), including two amino acids (Figure 1D, Table 1). Additionally, heat map and PLS-DA analysis achieved great separation among tumor and normal based on the 10 metabolites (Figures 1E,F). As many literatures have reported that amino acid metabolism reprogramming is involved in tumor development, thus, our study mainly focused on OSCC amino acid metabolism (4). Receiver operating characteristic curve analysis of all metabolites revealed 10 amino acids were selected as candidate metabolic biomarkers [area under the curve (AUC) >0.80; Figure 2, Table 2]. Four of 10 amino acids had or close statistical significance [glutamate, serine, γ-aminobutyric acid (GABA), alanine], and the other six amino acids had no statistical significance (*t*-test), which may be related to our small samples.

**Table 1:** Differential metabolites identified by *t*-test between tumor and normal tissues.

**Metabolites**	**FC**	**log_**2**_(FC)**	***P***	**–log_**10**_(p)**
Lactic acid	0.026484	−5.2387	0.01	1.992
Glutamate	1.8472	0.88538	0.011	1.9707
Cholesterol	1.5545	0.63648	0.012	1.9145
L-kynurenine	4.1458	2.0517	0.017	1.7685
Maltose	0.43077	−1.215	0.017	1.7681
Glyceric acid	0.40825	−1.2925	0.02	1.6933
Muconic acid	0.45291	−1.1427	0.026	1.5698
Thymine	2.1013	1.0713	0.034	1.4656
Uridine	2.1739	1.1203	0.039	1.4024
Gamma-aminobutyric acid	2.1963	1.1351	0.048	1.3124

**Table 2:** AUC values of potential OSCC biomarkers by ROC curve analysis in development and validation group.

	**Untargeted group**		**Targeted group**	
**Metabolite**	**AUC**	***P***	**AUC**	***P***
Glutamate	0.844	0.011	0.935	<0.0001
Aspartic acid	0.812	0.112	0.918	<0.0001
Proline	0.812	0.1	0.915	<0.0001
Tyrosine	0.844	0.289	0.825	0.001
Serine	0.812	0.068	0.822	0.0022
Threonine	0.812	0.167	0.816	0.0009
GABA	0.828	0.048	0.792	0.0026
Lysine	0.859	0.163	0.78	0.0035
Alanine	0.828	0.062	0.68	0.0405
Isoleucine	0.828	0.369	Not detected	Not detected

*ROC, receiver operating characteristic; P, P-values of t-test*.

### Identification of Three Amino Acids as OSCC Diagnostic Biomarkers by UPHLC-MS Targeted Metabolomics Analyses

**Figure 3:**
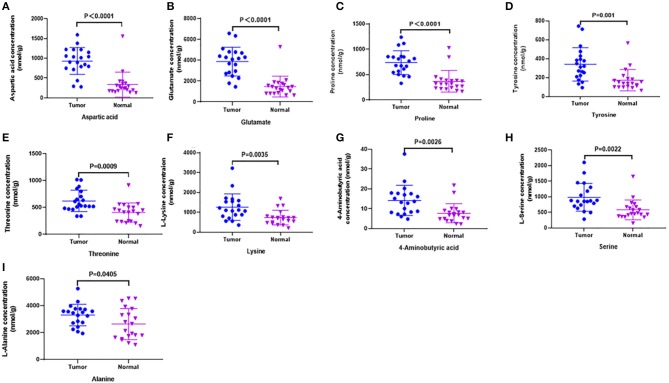
Candidate amino acids biomarkers (AUC > 0.80) identified by UHPLC-MS/MS quantitative analysis in another batch of 20 cases (targeted group). **(A–I)** The results showed 9 of 10 were consistent with untargeted group (threshold, *t*-test, *P* < 0.05), which had significant differences between tumor and normal.

**Figure 4:**
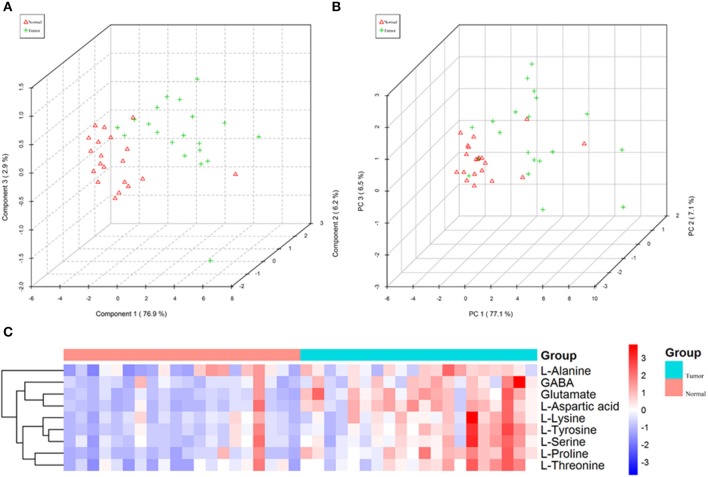
The nine amino acids identified by quantitative analysis can significantly distinguish tumors from normal tissues in the targeted group. **(A)** Scores plot accurately distinguishes tumor and normal by PLS-DA analysis in the targeted group. **(B)** Scores plot distinguishes tumor and normal by PCA analysis in the targeted group. **(C)** Comparison of tumor and normal using normalized intensities of nine significance metabolites in the targeted group. The mean value of nine metabolites in each group was obtained, and then *Z*-score transformation generates heat map.

**Figure 5:**
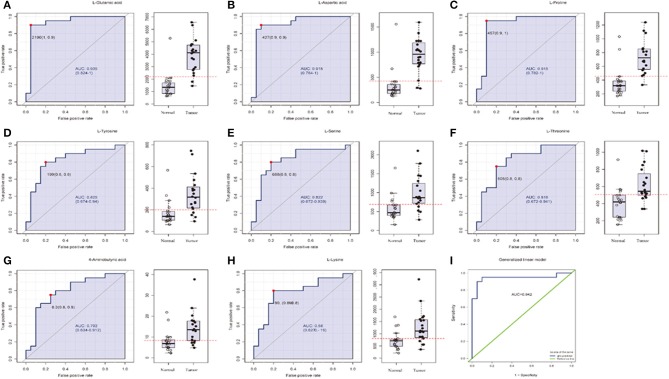
The list of three amino acids (AUC > 0.90) selected as potential tumor biomarkers by ROC curve analysis and binary logistic regression in targeted group. **(A–H)** Individual amino acid ROC curves (AUC > 0.70). **(I)** Analyses for three amino acids (aspartic acid, glutamate, proline, AUC > 0.90, *t*-test *P* < 0.0001) were performed by binary logistic regression in Glmnet package; optimal sensitivity and specificity in targeted group were obtained (AUC = 0.942). AUC (0.5–0.7), low accuracy; AUC (0.7–0.9), moderate accuracy; AUC (>0.9), high accuracy.

In order to test whether the 10 amino acids we selected could accurately distinguish tumors from normal tissues, we used another batch of 20 cases; each case contains paired tumor and normal tissues. The specific concentration of the targeted amino acids in all samples was examined by UHPLC-MS/MS quantitative analysis. The results showed nine metabolites were detected, which had significant differences (*t*-test, *p* < 0.05; Figure 3, Table 2); one was not detected. Principal component analysis, PLS-DA, and heat map analysis achieved great separation among tumor and normal groups based on the nine metabolites (Figure 4). We tested the efficiency of more than nine amino acids by ROC curve analysis; three of nine metabolites (glutamate, aspartic acid, and proline) displayed high efficiency (AUC >0.90); six of nine amino acid markers displayed moderate efficiency (AUC = 0.70–0.90; Figures 5A–H, Table 2). Moreover, we obtained optimal sensitivity and specificity using the top three amino acids (AUC >0.90, *p* < 0.0001) by binary logistic regression in the Glmnet package (AUC = 0.942; Figure 5I). These results suggest that the three amino acids (glutamate, aspartic acid, and proline) could be used as OSCC potential diagnostic biomarkers.

## Discussion

Metabolic change was one of the hallmarks of cancer cells, in which regulatory networks were altered to adapt to the metabolic pressures and provide energy for cancer cells growth, namely, metabolic reprogram (16, 17). Compared to other “omics” (such as proteome/genome/transcriptome), most metabolites are small molecular compounds, are highly conservative, and have stable performance. The statistical analysis of metabolomics data is more convenient; the results are easier to understand and more accurate. The study on metabolomics of cancer tissue specimens is helpful to improve the diagnosis and treatment of OSCC. Currently, metabolomics studies of oral cancer use primarily biological fluids (e.g., plasma, urine, saliva) and cell lines (18–22), because biofluids and cell lines are readily available. Most biofluids-based metabolomics studies are relatively quantitative, without in-depth study on a specific metabolic type and a specific metabolite, such as amino acid metabolism and the specific content of a certain amino acid. Therefore, we urgently need to use tumor tissue and normal tissue samples to conduct systematic studies on a certain subtype of metabolomics (such as amino acid metabolomics) and finally develop a panel of markers for the early diagnosis of OSCC.

In this study, we first performed GC-MS high-throughput untargeted analysis of OSCC tumor tissue and matched normal tissue samples, 10 differential metabolites were selected by *t*-test to distinguish tumors from normal tissue. Many literatures have reported that amino acid metabolism reprogramming is involved in tumor development; moreover, the quantitative analysis of amino acids is convenient. Therefore, we focused on the amino acid metabolism of OSCC. A total of 10 amino acids (such as glutamate, GABA, lysine, aspartic acid, tyrosine, serine, threonine, alanine, proline, isoleucine) were selected as candidate biomarkers by ROC curve analysis (AUC >0.80) in GC-MS untargeted group.

In order to further accurately determine the oncogenic metabolites that distinguish tumors from normal tissues, UHPLC-MS/MS targeted quantitative analysis was performed on another batch of 20 cases (each case contains tumor and normal tissues). We measured the specific concentration of the above 10 amino acids in all samples. The results validated that 9 of 10 were detected, which had statistical differences between tumor and normal (*t*-test, *p* < 0.05). Glutamate, aspartic acid, and proline displayed high sensitivity and specificity (AUC >0.90) by ROC curve analysis. Moreover, obtaining optimal sensitivity and specificity uses the three amino acids by binary logistic regression in the Glmnet package. These results suggest that the three amino acids (glutamate, aspartic acid, and proline) could be used as OSCC potential biomarkers. It had been reported that OSCC tumor tissues had higher amino acid levels than normal tissues (22, 23); our research has produced similar results. However, there were reports that revealed a lower relative concentration of amino acids as compared to healthy groups in some cancers, such as breast, pancreatic, oral, and colorectal cancers (24–26). This anomaly suggests that cancer cells build a second metabolic pathway to generate energy for rapid growth, which needs more glucogenic amino acids.

It is famously known that cancer cells favor the “Warburg effect,” that is, the enhanced glycolysis or aerobic glycolysis, even when the ambient oxygen supply is sufficient. In addition, deregulated anabolism/catabolism of fatty acids and amino acids, especially glutamine, serine, and glycine, has been identified to function as metabolic regulators in supporting cancer cell growth (7, 28, 29). In addition to being utilized as substrates for protein synthesis, amino acids have been documented by mounting studies that they function as metabolites and metabolic regulators in supporting cancer cell growth, among which research on glutamine, serine, and glycine has been focused (30). It also revealed that cancer cells undergo biosynthesis during proliferation, which requires a large amount of amino acids, resulting in an increase in the concentration of amino acids in cells during the synthesis process and a decrease in the concentration of amino acids at the end of synthesis. Altered cell metabolism enables tumors to sustain their increased energetic and biosynthetic needs. Our results suggest that the three amino acids (glutamate, aspartic acid, and proline) could be used as OSCC potential biomarkers and may be involved in the progression of OSCC.

However, because of the limited size of the patients, our study is a preliminary study, and the accuracy of the diagnostic values of our identified panels (glutamate, aspartic acid, and proline) remained to be verified by more samples. The molecular mechanism of amino acid metabolism promoting OSCC and related metabolic pathways deserve further study.

## Conclusion

The results from this study were based on GS-MS untargeted metabolomics analysis and UHPLC-MS targeted quantitative analysis revealing three amino acids (glutamate, aspartic acid, and proline) as potential biomarkers of OSCC.

## Data Availability Statement

All datasets generated for this study are included in the article/supplementary material.

## Ethics Statement

This study was reviewed and approved by the medical ethics committee of Nanjing Stomatological Hospital, Medical School of Nanjing University, and carried out according to the recommendations of the Declaration of Helsinki. All cases included in the study were between 30 and 70 years old and had signed informed consent forms.

## Author Contributions

X-HY, Q-GH, and Y-HN: investigation and project administration. SC and LZ: software application. X-HY, FD, and SW: data analysis. X-XZ and YJ: methodology and supervision. Y-HN and YJ: validation data. X-HY and Y-HN: wrote the draft. Q-GH: writing-review and editing.

### Conflict of Interest

The authors declare that the research was conducted in the absence of any commercial or financial relationships that could be construed as a potential conflict of interest.
